# Draft Genome Sequence of *Bifidobacterium aesculapii* DSM 26737^T^, Isolated from Feces of Baby Common Marmoset

**DOI:** 10.1128/genomeA.01463-15

**Published:** 2015-12-10

**Authors:** Hidehiro Toh, Yumiko Yamazaki, Kosuke Tashiro, Shinpei Kawarai, Kenshiro Oshima, Akiyo Nakano, Co Nguyen Thi Kim, Iyo Mimura, Kensuke Arakawa, Atsushi Iriki, Takefumi Kikusui, Hidetoshi Morita

**Affiliations:** a Medical Institute of Bioregulation, Kyushu University, Fukuoka, Japan; b Riken Brain Science Institute, Wako, Japan; c Graduate School of Systems Life Sciences, Kyushu University, Fukuoka, Japan; d School of Veterinary Medicine, Azabu University, Sagamihara, Kanagawa, Japan; e Graduate School of Frontier Sciences, The University of Tokyo, Kashiwa, Chiba, Japan; f Department of Microbiology and Infectious Diseases, Nara Medical University, Kashihara, Nara, Japan; g Graduate School of Environmental and Life Science, Okayama University, Okayama, Japan

## Abstract

*Bifidobacterium aesculapii* DSM 26737^T^ was isolated from feces of baby common marmoset. Here, we report the draft genome sequence of this organism. This paper is the first published report of the genomic sequence of *B. aesculapii*.

## GENOME ANNOUNCEMENT

Bifidobacteria are high-G+C-content Gram-positive bacteria that are commonly found in the human and animal gastrointestinal tracts. Bifidobacteria are widely used as probiotic organisms, which confer a health benefit to the host when administered in adequate amounts. Genome sequences of bifidobacterial strains residing in the human gastrointestinal tract have been determined (1). However, studies of bifidobacteria of nonhuman primates are very few. Novel species within the genus *Bifidobacterium* isolated from common marmoset (*Callithrix jacchus*) have recently been reported (2–4). *Bifidobacterium aesculapii* DSM 26737^T^ (= JCM 18761^T^) was isolated from fecal samples of baby common marmoset (3). *B. aesculapii* DSM 26737^T^ is related to *Bifidobacterium stellenboschense* DSM 23968^T^, which was isolated from feces of tamarin (red-handed marmoset) (2), in the phylogenetic tree of the genus *Bifidobacterium* (3).

The *B. aesculapii* DSM 26737^T^ genome was paired-end sequenced using Illumina’s MiSeq platform. Genomic libraries containing 600 to 1,000 bp inserts were constructed and sequenced, yielding 3,782,020 sequences that provided 418-fold coverage from both ends of the genomic clones. The sequence reads were assembled using Newbler version 2.8 (Roche), and the assembled genome consists of 93 contigs with a total length of 2,693,486 bp. The genome has a G+C content of 64.8%, which is the higher G+C content in the *Bifidobacterium* species. The genome size was larger than those of bifidobacterial strains residing in the human gut. The draft genome of *B. aesculapii* DSM 26737^T^ contained 2,070 predicted protein-coding genes and 58 tRNA genes. Then, we compared the draft genome of DSM 26737^T^ with that of *B. stellenboschense* DSM 23968^T^ (accession no. JGZP01000000) (5). Of the 2,070 protein-coding genes, 1,486 (72%) were shared by the both strains. The genome information of this species will be useful for further studies of its physiology, taxonomy, and ecology.

### Nucleotide sequence accession numbers.

The draft genome sequence for *B. aesculapii* DSM 26737^T^ has been deposited in the DDBJ/GenBank/EMBL database under the accession numbers BCFK01000001 to BCFK01000093.
